# Metabolic Dysfunction in Motor Neuron Disease: Shedding Light through the Lens of Autophagy

**DOI:** 10.3390/metabo12070574

**Published:** 2022-06-22

**Authors:** Subhavi De Silva, Bradley J. Turner, Nirma D. Perera

**Affiliations:** Florey Institute of Neuroscience and Mental Health, University of Melbourne, Parkville, VIC 3052, Australia; desilva.s@florey.edu.au (S.D.S.); bradley.turner@florey.edu.au (B.J.T.)

**Keywords:** amyotrophic lateral sclerosis, metabolism, autophagy, energy homeostasis, protein degradation, lipids, superoxide dismutase 1 (SOD1)

## Abstract

Amyotrophic lateral sclerosis (ALS) patients show a myriad of energetic abnormalities, such as weight loss, hypermetabolism, and dyslipidaemia. Evidence suggests that these indices correlate with and ultimately affect the duration of survival. This review aims to discuss ALS metabolic abnormalities in the context of autophagy, the primordial system acting at the cellular level for energy production during nutrient deficiency. As the primary pathway of protein degradation in eukaryotic cells, the fundamental role of cellular autophagy is the adaptation to metabolic demands. Therefore, autophagy is tightly coupled to cellular metabolism. We review evidence that the delicate balance between autophagy and metabolism is aberrant in ALS, giving rise to intracellular and systemic pathophysiology observations. Understanding the metabolism autophagy crosstalk can lead to the identification of novel therapeutic targets for ALS.

## 1. Motor Neuron Disease (MND)

MND is an umbrella term describing a group of progressive neurological disorders affecting motor neurons. The most common MND is ALS, which is an adult-onset disease that causes progressive muscle weakness and paralysis, eventually leading to respiratory failure and death [1]. ALS affects the upper motor neurons that project from the motor cortex of the brain to the brainstem and spinal cord and lower motor neurons that originate in the spinal cord that innervate voluntary muscles. This pathology results in symptoms, such as spasticity, muscle weakness, muscle wasting, cramps, fasciculations, paralysis, and pseudobulbar symptoms [2]. While the early manifestations of bulbar-onset ALS include dysarthria (difficulty speaking) and dysphagia (difficulty swallowing), most patients gradually develop these conditions as the disease progresses [3]. 

ALS can be categorized into two classes depending on causation: familial ALS caused by inherited gene mutations responsible for approximately 10% of ALS and sporadic ALS lacking a family history responsible for approximately 90% of cases [4]. The two Food and Drug Administration (FDA)-approved treatments for ALS both have very modest effects on disease progression. Riluzole modulates glutamatergic neurotransmission and increases survival by approximately 3–4 months [5,6] and edaravone, a free radical scavenger, reduces oxidative stress and has been shown effective in a limited subset of ALS patients who satisfy certain selection criteria [7,8]. 

The first genetic mutation identified in ALS was the superoxide dismutase 1 (SOD1) gene [9]. Many others have since been identified (Table 1), which can be broadly classified into three categories based on their functions: protein homeostasis, RNA-binding/metabolism, and cytoskeletal dynamics [10]. It is important to note that overlap exists between these mechanisms [10].

As indicated in Table 1, the high genetic heterogeneity makes ALS a complex disease to unravel. However, a common thread in ALS pathology is the presence of protein aggregates in affected motor neurons and glia [25]. Specifically, mis-localised, phosphorylated TDP-43 is frequently found within cytoplasmic inclusions inside neurons and glia of ALS patients [26,27]. TDP-43 aggregates are present in ~97% of patients [28], pointing to the dysfunction of protein degradation pathways as a core pathological process in ALS. There are two main protein degradation pathways in eukaryotic cells: ubiquitin proteasome system and autophagy. Autophagy is the primary intracellular catabolic pathway that can degrade large protein aggregates and damaged organelles in the cytoplasm [29].

## 2. Autophagy: A Brief Overview

Three types of autophagy have been identified: microautophagy, chaperone-mediated autophagy, and macroautophagy. Microautophagy involves lysosomes directly engulfing components in the cytoplasm through invagination of the lysosomal membrane [30]. Chaperone-mediated autophagy degrades specific proteins with the aid of the chaperone protein heat shock cognate 71 kDa (HSC70) and lysosomal-associated membrane protein 2A (LAMP2A) [31]. The key autophagy pathway that degrades the bulk of cytoplasmic components, however, is macroautophagy. Importantly, the clearance of large protein aggregates relies exclusively on macroautophagy [32]. Therefore, the term macroautophagy (hereafter referred to as autophagy) is used synonymously in the field with the word autophagy. 

Autophagy degrades cytoplasmic cargo by sequestering unwanted cellular material into double-membrane-bound structures called autophagosomes. Autophagosomes eventually fuse with lysosomes containing digestive enzymes, forming autolysosomes [33]. Inside the autolysosome, cytoplasmic components are enzymatically degraded, resulting in the release of monosaccharides, amino acids, fatty acids, and nucleic acids back into the cytoplasm. Motor neurons in sporadic ALS patients show accumulation of autophagosomes and autolysosomes, which are often closely associated with skein-like inclusions, Bunina bodies, and round bodies characteristic of ALS, indicating autophagy impairment [34]. Indeed, there is well-documented evidence for autophagy dysfunction in ALS patients and mouse models (reviewed in [35]).

Basal autophagy plays a critical role in the recycling of nutrients and the maintenance of cellular homeostasis [36] and has an essential role in the lifespan and health span of mammals [37,38]. In times of cellular stresses, such as hypoxia, nutrient deprivation, and starvation, autophagy is activated to promote cell survival [39,40]. Thus, autophagy is tightly regulated by the mammalian target of rapamycin complex 1 (mTORC1) and AMP-activated protein kinase (AMPK), which are key upstream regulators of the pathway due to their involvement in metabolite and energy sensing, respectively [41]. Complex autophagy signalling is highly regulated by approximately 20 core autophagy-related genes (ATGs), which encode proteins involved in autophagosome formation (reviewed in [42,43]). The functions of the core autophagy genes that are discussed in this review are listed in Table 2.

As a process critical for maintaining intracellular homeostasis, autophagy is fundamentally linked to cellular energy metabolism.

## 3. Metabolism: A Brief Overview

Metabolism refers to the collection of chemical reactions that occur in the body to sustain life and can be broadly divided into three categories: anabolism, catabolism, and elimination of wastes [41]. Anabolic reactions use energy to synthesize complex molecules, such as the synthesis of glycogen from glucose. Catabolic reactions break down large molecules releasing energy and include cellular respiration and autophagy [41]. It should be noted that the energy metabolism of the CNS differs markedly from the rest of the body. The brain has a high energy demand and consumes 20% of the oxygen and 25% of glucose [51]. Neuronal energy metabolism is complex in that neurons can use alternative energy substrates, such as lactate, pyruvate, and glutamate, in addition to glucose [52]. To achieve this, neurons rely on intricate interactions with astrocytes. Astrocytes are highly glycolytic and break down glucose into lactate, which is subsequently released into the extracellular space and taken up by neurons through a lactate shuttle [51]. 

There is expanding evidence of multitudes of metabolic abnormalities in ALS (reviewed in [53]). Weight loss, which is not always attributable to dysphagia, is a common observation in patients [54,55], as is hypermetabolism, characterized by an increased basal metabolic rate [56,57]. It has been suggested that weight loss could occur if the increased energy expenditure in hypermetabolic patients is not balanced by an increase in dietary intake [58]. However, a direct causational link between weight loss and hypermetabolism has not yet been identified. Interestingly, one study found no significant reduction in weight or BMI in hypermetabolic ALS patients compared to normometabolic patients [59]. Rapid weight loss is often synonymous with poor prognosis and reduced survival [60,61]. A low body mass index (BMI) indicating malnutrition similarly causes poor prognosis and reduced survival in ALS [62]. 

## 4. Relationship between Autophagy and Metabolism—Implications for MND

The catabolism caused by autophagy resulting in simple monosaccharides, fatty acids, and amino acids can be used to generate energy via ATP or provide building blocks for essential protein synthesis [63]. Hence, as a process critical for maintaining cellular energy homeostasis, autophagy is fundamentally linked to metabolism and acts as a powerful promoter and regulator of metabolism, at both the cellular and organismal level [63,64]. The critical role autophagy plays in regulating cellular metabolic capabilities is evident in autophagy dysregulation, underpinning metabolic disorders, such as insulin resistance, diabetes mellitus, obesity, atherosclerosis, and osteoporosis [65]. 

Analogous to the CNS energy metabolism being distinct from the other organs, evidence indicates that the modulation of autophagic activity is also tissue specific [65]. Indeed, the level of autophagy activity and induction is unique in neurons that are highly polarised [66]. Autophagic clearance of proteotoxins is critical for post-mitotic neuronal survival [67,68]. Therefore, it should be noted that the complex inter-relationship between autophagy and metabolism is cell- and organ-specific (metabolic effects of various tissue-specific autophagy gene deletions are summarized in Table 3).

Motor neurons are especially vulnerable to metabolic and autophagic defects as they have a high energy demand to sustain their large size and long axons. Given the multi-systemic nature of MND, the following sections discuss the role of autophagy in the metabolism of nutrients within the context of various cell types and how this relationship is altered in MND patients and mouse models.

## 5. Autophagy and Carbohydrate Metabolism

### 5.1. Carbohydrate Metabolism—The Basics

Glucose is transported into the cell through the glucose transporter (GLUT) family and sodium glucose cotransporters (SGLTs) via facilitated diffusion [83]. This is followed by quick phosphorylation to form glucose 6-phosphate to essentially trap glucose within the cytoplasm [83]. Glucose 6-phosphate is considered a central molecule of metabolism as it can be utilised in multiple pathways depending on cellular conditions and metabolic needs [83]. These include glycolysis, gluconeogenesis (which occurs in the liver and during starvation in kidneys to form glucose again)*,* glycogenesis (storage as glycogen), or the pentose phosphate pathway (to generate NADPH for fatty acid synthesis) [83]. 

### 5.2. Autophagy and Glycolysis

Glycolysis is the first major eukaryotic energy-producing process in the cell, leading to the formation of pyruvate and lactate [83]. Under aerobic conditions, pyruvate is transported to mitochondria and converted to acetyl CoA, which enters the citric acid cycle, the second major energy-producing process [83]. The eight-step citric acid cycle generates NADH that can then be utilised in the third energy pathway, the electron transport chain in oxidative phosphorylation producing ATP [83]. Under normal circumstances, neurons have a lower rate of glycolysis in contrast to astrocytes, which have higher basal glycolysis rates and lactate production [84]. Indeed, neurons use glycolysis as a fast mechanism to generate ATP from glucose [85]. Particularly during increased energy demand, nerve terminals rely heavily on glycolysis for synaptic function, fast axonal transport, and meeting local energy demands in pre-synaptic nerve terminals [85]. 

Sugars, including glucose, released following autophagic breakdown are catabolized by glycolysis [64]. Therefore, the changes in enzymatic activity of glycolysis-related enzymes, such as hexokinase and pyruvate kinase, associate with autophagy and affect the autophagy pathway [63]. Key evidence that autophagy is required for glucose metabolism was gained when the essential autophagy gene *ATG7* was conditionally deleted in adult mice [77]. This acute whole-body *ATG7* deletion gave rise to a systemic autophagy blockade without extensive organ damage 5 weeks after deletion [77]. In contrast, by 6–12 weeks following deletion, extensive liver and muscle damage was evident. Finally, neurodegeneration limited the survival of these mice to 2–3 months [77]. Upon fasting, mice displayed extreme muscle wasting and died of hypoglycemia, indicating the essential role autophagy plays in glucose homeostasis [77]. Furthermore, mice with β cell-specific *ATG7* gene deletion showed low insulin secretion accompanied by hypoinsulinemia and impaired glucose tolerance [81,82]. This suggests that autophagy is necessary to maintain the structure, mass, and function of pancreatic β cells, its impairment causing insulin deficiency and hyperglycemia [82]. Further, diabetes induced by ER stress was shown to be alleviated by increasing autophagy degradation [41]. This suggests that insulin release is associated with an autophagy-regulated secretion mechanism [41]. These findings imply that autophagy not only regulates systemic glucose metabolism upon nutrient starvation but also controls the overall physiological glucose metabolism via regulating pancreatic insulin secretion.

#### Implications for MND

SOD1^G93A^ hemizygous transgenic mice expressing a high copy number of the human *SOD1G93A* mutation are the most widely used ALS model as they recapitulate ALS symptoms and progression [86]. SOD1^G93A^ mice show CNS impairments in pyruvate production and conversion toward lactate and alanine, decreased gene expression of the glycolytic enzymes, and reduced glucose entry into the TCA cycle indicating defects of glycolysis (reviewed in [85]). Additionally, activation of glycolysis via GLUT3 and phosphofructokinase overexpression in motor neurons was neuroprotective and improved locomotion in a TDP-43 Drosophila model, suggesting that glycolysis activators could slow disease progression and prolong survival in ALS [85]. In ALS skeletal muscle, a selective loss of glycolytic fibers and reduction in the activity of phosphofructokinase, a key enzyme involved in glucose metabolism, were observed early in the pre-symptomatic stage [87]. This was accompanied by a metabolic transition towards oxidative metabolism with an increase in mitochondrial mass at disease onset [88,89]. 

How these glycolytic impairments directly affect autophagy or the cross-talk between the two pathways in the context of ALS remains currently unknown. However, one study investigated the effects of metformin, a drug that regulates both glucose metabolism and autophagy, which resulted in some interesting findings in the SOD1^G93A^ mouse model. As an anti-type 2 diabetic drug, metformin reduces hepatic glucose production, increases insulin sensitivity, and activates autophagy through the AMPK-mTOR signalling axis. However, metformin was unable to reduce pathology in SOD1^G93A^ mice and had an unexpected negative effect on the symptom onset and disease progression in females [90]. These negative effects of metformin were attributed to effects on mitochondrial complex I and total and LDL cholesterol levels. Metformin was reported to inhibit mitochondrial complex one [91], which is already compromised in ALS, and reduce total and LDL cholesterol levels when high levels of these markers are a positive prognostic factor in ALS patients [92]. It should also be noted here that pre-morbid diabetes mellitus is reported to have a positive effect on age of ALS onset [93]. These results suggest that more targeted approaches are required to modulate the intricate glucose metabolic deficits observed in ALS.

### 5.3. Autophagy and Oxidative Phosphorylation

Mitochondrial oxidative phosphorylation is the most important source of ATP [63]. Neurons rely mainly on oxidative phosphorylation to fuel their high metabolic demands, and any deviation from this norm would lead to neurological disorders [94]. The integrity of the inner mitochondrial membrane, particularly mitochondrial respiratory chain activity, is important for determining autophagy activity [63]. Mitochondrial respiratory defects significantly reduce autophagy levels in yeast and mammalian cells [63]. Mitochondrial inhibitors, on the other hand, led to a decrease in ATP levels, activation of AMPK, inactivation of mTOR, and induction of autophagy [63]. Specifically, mitochondrial complexes I, III, and IV act as important “tuners” that can positively regulate basal and induced autophagy and are key molecules linking basal autophagy and the cellular energy flux, suggesting that there is a tight correlation between oxidative phosphorylation in the mitochondrial respiratory chain and autophagy [63,95].

#### Implications for MND

A plethora of studies has shown mitochondrial abnormalities in the CNS of ALS patients and mouse models [96,97,98,99,100,101,102]. Specifically, impaired electron transport chain activities, reduced oxidative phosphorylation, and subsequent reductions in ATP generation have been reported [97,102,103,104,105]. In line with this, restoring NAD+, which is an essential substrate for oxidative phosphorylation, was found to improve survival in SOD1^G93A^ mice, as well as improve ALS functional rating score, pulmonary function, and muscular strength in a small cohort of patients [85]. Both the relative abundance of NAD+ (oxidised form) and NADH (reduced form) and the total NAD content plays an important role in autophagy regulation [63,106]. 

Studies suggest that structural and functional mitochondrial abnormalities, leading to impaired glucose metabolism and generation of ATP, might be caused by protein aggregation within mitochondria (reviewed in [85]). There is a wealth of evidence that mutant TDP-43 [107], overexpressed mutant human FUS [108], and mutant SOD1 [109,110] accumulate inside the mitochondria of ALS patients, Drosophila models, and SOD1 mouse brain tissue, respectively, and cause defective mitochondrial complex activities, impaired protein homeostasis and reduced mitochondrial ATP production [111,112].

The autophagy sub-type that selectively sequesters and eliminates damaged or aged mitochondria is called mitophagy. In this mitochondrial quality control pathway that is essential for neuronal homeostasis, the E3 ubiquitin ligase parkin targets damaged mitochondria for degradation by autophagosomes [113]. Evidence that mitophagy is dysfunctional in ALS comes from the discovery that familial ALS genes *OPTN* (ubiquitin-binding autophagy receptor/adapter) and *TBK1* (kinase known to phosphorylate p62 and OPTN) regulate mitochondrial turnover [114,115,116]. Inefficient turnover of mitochondria due to ALS-associated mutants could be a contributing factor leading to the accumulation of dysfunctional mitochondria in ALS motor neurons (reviewed in [117]). For example, the expression of loss of function mutants from *OPTN* or *TBK1* resulted in impaired mitophagy and accumulation of damaged mitochondria in Hela cells [113,118]. The use of an ALS-linked TBK1 mutant significantly reduced OPTN and LC3B recruitment to damaged mitochondria, an observation that was similar to silencing *TBK1* or using a potent TBK1 inhibitor [118,119]. However, it is essential to balance the mitophagic clearance of damaged mitochondria with mitochondrial biogenesis in ALS. This was exemplified by the effects of the autophagy inducer rilmenidine in SOD1^G93A^ [120] and TDP-43 [121] mice, where excessive mitochondrial clearance led to an acceleration in disease progression. 

### 5.4. Autophagy and Oxidative Stress

Mitochondrial dysfunction and oxidative stress are directly interlinked where inefficient mitochondrial oxidative phosphorylation causes the accumulation of reactive oxygen species (ROS) that causes oxidative stress by damaging intracellular DNA, lipids, and proteins, leading to necrosis and apoptotic cell death [122]. In this context, unfolded protein aggregates formed by oxidative stress lead to the activation of autophagy [123] where autophagy plays a protective role to limit the damage from ROS [63]. To benefit cell survival, induced autophagy degrades aggregates of damaged mitochondria and reduces ROS-induced damage, subsequently preventing cell death [63,124]. However, it should be noted that proteolysis by autophagy itself could be a potential source of free radicals [122].

#### Implications for MND

Oxidative-stress-mediated protein injury, lipid peroxidation, and DNA and RNA oxidation have been observed in ALS patients, with oxidative stress biomarkers profoundly found in sporadic ALS patient urine, cerebrospinal fluid (CSF), and blood [122]. Indeed, edavarone is a free radical scavenger that removes lipid peroxide and hydroxyl radicals via transferring electrons to the radicals, leading to neuroprotection [122]. It is important to note that the function of SOD is to convert oxygen radicals (O_2_) to hydrogen peroxide (H_2_O_2_), which is further reduced by catalase or peroxidase to form oxygen and water [63]. In this context, hydrogen peroxide is a relatively stable signal regulating autophagy [63]. Hydrogen peroxide stabilizes lipidated LC3 and gamma aminobutyric-acid-receptor-associated protein-like 2 (GABARAPL2) by directly oxidizing ATG4 [63]. The hypothesis that mutant SOD1 causes ALS disease pathology by a gain of function rather than a loss of function mechanism [11] suggests that intricate signalling between oxidative stress pathways and autophagy is likely impaired by mutations in the SOD1 protein. 

## 6. Autophagy and Lipid Metabolism

Autophagy and lipid metabolism share regulatory and functional similarities as both pathways are activated following nutrient deprivation [125]. The former breaks down redundant cellular components to provide molecules critical for cell survival and the latter for mobilization of cellular lipid stores to supply free fatty acids for energy. Therefore, crosstalk exists between lipid metabolism and autophagy [64]. Lipids regulate autophagy in several ways. Firstly, lipid-derived metabolites activate mTOR to downregulate autophagy initiation [63]. Secondly, lipid molecules mediate the double-membrane structure of autophagy vesicles, by mediating morphological changes that occur during the formation and maturation of autophagosomes, by regulating lipidation of ATG8/LC3 family proteins (i.e., covalently binding to PE) via promoting the extension of the phagophores and the formation of autophagosomes, or by controlling the distribution of lipid species in the double-membrane structure [63].

Intracellular lipids, except those membrane bound, are mainly present in the form of lipid droplets [63]. Lipid droplets are ubiquitous fat-storage organelles and play key roles in lipid metabolism and energy homeostasis [126]. These ubiquitous organelles have a unique structure with a core of neutral lipids (triacylglycerols and sterol esters) surrounded by a monolayer of phospholipids and proteins [126]. The triacylglycerols and cholesterol within the lipid droplets can be degraded by β-oxidation in mitochondria to provide ATP [63]. Lipid droplets originate from the ER and, when lipids are needed, they are turned over by cytoplasmic lipases and autophagy in a specific autophagy type termed lipophagy [63,126]. Lipophagy regulates lipid metabolism in three ways. Firstly, by blocking autophagy via increasing the amount of lipids in lipid droplets. Secondly, through the SNARE complex, which mediates the fusion of lipid droplets and autophagosomes using the LC3 localized on lipid droplets. Thirdly, by ATG15 acting as an esterase [63]. Hence, abnormalities in lipophagy lead to lipid metabolism disorders, such as obesity, arteriosclerosis, and diabetes [63]. Further, emerging evidence indicates that lipid droplets have functions beyond energy storage, such as the resolution of ER stress, contribution to protein storage, folding, and the clearing of protein aggregates [127,128].

### 6.1. Lipid Droplets in CNS

In the brain, lipid droplets are found mainly in glial cells and help to provide fuel for neurons when glucose is scarce in times of energy need [129]. Neuronal lipid droplets are rare, even in diseased states [126]. However, in the aged brain, lipid droplets were observed in neurons in addition to microglia, astrocytes, and ependymal cells [130]. Emerging evidence suggests that lipid droplets play a fundamental role in protein aggregation and clearance [126]. Lipid droplets colocalize with the autophagy marker LC3, which may indicate that lipophagy is used to degrade lipid droplets in the brain [130]. It is reported that chaperone-mediated autophagy decreases with age [131,132]. If lipophagy also decreases with age, this could contribute to the lipid droplet accumulation observed with age [133].

#### Implications for MND

Lipid droplets were shown to accumulate in glial cells surrounding motor neurons in SOD1^G93A^ rats [134,135]. SOD1^G93A^ rats displayed drastic elevation of hydroxylated ceramides at the symptomatic stage [134], where the accumulation of hydroxylated ceramides was shown to trigger autophagy [136]. As such, increased autophagy was shown in these glial cells of SOD1 rats, as indicated by increased LC3-II immunoreactivity, which was particularly abundant around the lipid droplets [135].

It was proposed that glial lipid droplets sequester lipids away from the plasma membrane, especially lipids containing polyunsaturated fatty acids (PUFAs), which are particularly vulnerable to peroxidation by ROS [126]. Consistently, markers of lipid peroxidation are found in the CSF of ALS patients and disease models, suggesting that the accumulation of these toxic molecules may contribute to ALS [126,137,138]. PUFAs are more protected when incorporated into triacylglycerols within lipid droplets than when they are in the phospholipids of cell membranes [126]. During ROS-induced apoptosis, PUFAs of membrane phospholipids are peroxidated, whereas their incorporation into lipid droplets is thought to decrease their toxicity, suggesting that protection from oxidative stress is a general role of lipid droplets [126,139]. This redistribution of fatty acids may, in turn, influence the lipid molecules available for the formation of double-membrane-bound autophagy vesicles and their LC3 lipidation, thereby affecting the level of autophagy in ALS.

ALS causative gene VAMP-associated protein B (*VAPB*)-induced neurotoxicity is linked to lipid droplet dynamics by the modifiers, such as acyl-CoA synthetase long-chain [140]. Further, the gain of function mutations in the lipid droplet protein seipin contributed to motor neuron disease symptoms in mice [140,141]. In WT mice, intracellular lipid accumulation was followed by lipid clearance from lysosomes into droplets [142]. However, when the gene *SPG11*, whose mutations contribute to an early-onset form of ALS, is knocked out in mice, there is a significantly slower rate of lysosomal lipid clearance and a decrease in lipid droplet size and number [142]. Together these studies suggest that impaired lipid droplet biogenesis may be an important pathological aspect of several ALS mutations [143].

TDP-43-overexpressing mice also display increased fat accumulation and adipocyte hypertrophy [144]. Depleting TDP-43 postnatally in mice causes weight loss, body fat reduction, decreased adipocyte lipid droplet content, increased fatty acid consumption, and rapid death [145]. Further, another study found that loss of C9orf72 leads to an overactivation of starvation-induced lipid metabolism that is mediated by dysregulated autophagic digestion of lipids and increased de-novo fatty acid synthesis [146]. This was mediated by coactivator-associated arginine methyltransferase (CARM-1), which, in turn, regulates autophagy–lysosomal functions and lipid metabolism [146]. In ALS/FTD patient-derived neurons or tissues, a reduction in C9orf72 function is associated with a dysregulation in the levels of CARM1, fatty acids, and NADPH oxidase NOX2 [146]. Taken together, these results suggest that the fine balance that exists between lipid homeostasis and autophagy is impaired in ALS, which may underlie the pathological and physiological symptoms observed.

### 6.2. Lipid Droplet and Autophagy Regulation of Motor Neuron Health

Since neuronal lipid droplets may be transient and/or difficult to detect, it is unclear if defects in lipid droplet homeostasis within motor neurons are a major driver of disease [133]. Therefore, the relevance of lipid droplet regulation to motor neuron health remains unknown [133]. However, it is important to note that fatty acid metabolism is coupled in neurons and astrocytes to protect neurons from fatty acid toxicity during periods of enhanced activity [147]. Hyperactivity and oxidative stress cause neurons to generate excess toxic fatty acids that are transferred via lipid particles to astrocytes where these fatty acids are detoxified as a means of protecting neurons [147]. Times of excitotoxicity are predicted to trigger lipid and fatty acid peroxidation [148,149], leading neurons to respond through activating autophagy to break down organelles damaged by ROS [147]. For example, peroxidated lipids in membranes of excited neurons are mobilized by autophagy for eventual removal from these cells [147]. Indeed, neuronal hyperactivity and hyperexcitability are important features of motor neuron disease where cortical hyperexcitability is observed as an early feature in ALS [150].

### 6.3. Lipid Metabolism Systemic Effects in MND 

Observational studies showed that ALS patients frequently have dyslipidemia [151]. Dyslipidemia is characterized by abnormal levels of high-density lipoprotein (HDL), low-density lipoprotein (LDL), total cholesterol, and triglycerides [151]. However, conflicting results have been reported for basal serum lipid levels, the cause of dyslipidemia, and the relationship between serum lipid levels and ALS disease progression [151]. Some studies report that most ALS patients [152] and mutant SOD1 mice [153,154] show abnormally low levels of lipids in the blood in a condition termed hypolipidemia—which is mainly characterized by low LDL levels. Hypolipidemia was shown to precede clinical symptom onset in mutant SOD1^G93A^ mice [153]. Whether hypolipidemia is also a preclinical feature in human ALS patients is difficult to assess, since diagnostic certainty is only reached in a progressed stage of the disease [84]. Elevated serum cholesterol and apolipoprotein E levels were shown to prolong survival and delay disease progression in ALS patients in most studies [92,155,156], while statin treatment was associated with worsened outcomes [157]. Interestingly, recent studies have shown that statins can modulate autophagy [158,159] where it was reported that the cytotoxic effect of simvastatin in NSC34 cells expressing mutant SOD1^G93A^ was mediated by the accumulation of autophagic vacuoles with elevated levels of LC3 II/I and P62, leading to impaired autophagic flux [160]. 

## 7. Switch from Glucose to Fatty Acid Usage in ALS 

### 7.1. CNS Glucose Utilisation: The Basics

Although neurons have extremely high energy demands, the brain lacks fuel stores [126]. When glucose is limited, neurons use energy-rich substrates, ultimately derived from the breakdown of fatty acids and, thus, relying on a continuous supply of glucose from peripheral organs, especially the liver [126]. When glucose is scarce, the liver converts acetyl-CoA produced during fatty acid β-oxidation into ketone bodies [126]. Ketone bodies are transported through the bloodstream to the brain, where they are oxidized to produce energy [126]. Fatty acids metabolized in the liver are, in turn, replenished from dietary lipids in the gut or from lipids stored in adipose tissue via lipolysis or lipophagy [126]. In all these tissues, fatty acids are stored as triacylglycerols in lipid droplets. Therefore, lipid droplet dysfunction could indirectly impact motor neurons, which is an attractive notion, as many ALS genes are ubiquitously expressed with non-cell-autonomous processes thought to contribute to ALS neurodegeneration [126].

### 7.2. Switch in ALS

Overall, evidence indicates that glucose metabolism is downregulated in ALS. In SOD1^G93A^ mice, the transition from glucose to lipid metabolism is evident by increased dependence and capacity of lipid mobilization demonstrated in the early phase of disease as compensation for mitochondrial dysfunction and defective glucose utilization [87]. This metabolic shift is further exacerbated by the severe impairments in glucose oxidation as the disease progresses [88,89]. However, it is still contentious whether this metabolic shift occurs at the level of the whole body early in the disease process. At least in skeletal muscle, this shift is observed early, prior to disease onset and before the activation of muscle denervation [89]. Elevated fatty acid consumption and oxidation by skeletal muscles may explain increased fat mobilization from adipose tissue [145,161]. Consistent with a change in fuel preference in SOD1 mice, denervation of glycolytic muscle fibers, an early pre-symptomatic event, is preceded by increased expression of lipid-handling genes [88,126]. 

In the spinal cord, in-vivo capillary imaging measuring spinal blood flow (SBF) and glucose metabolism showed early and progressive reduction in glucose usage [162]. However, spinal cord mitochondrial defects were only detectable at the symptomatic stage [87]. This has led to the suggestion that given the role of skeletal muscle in energy homeostasis, metabolic alterations in muscle occur independently of neuropathology and may underlie early pathological events in ALS [87,89]. This is further supported by the studies that report a fat-rich diet restores normal body mass, delays disease onset and motor neuron degeneration, and extends life expectancy in ALS [126,163]. Increased fatty acid requirements may explain why hyperlipidaemia positively correlates with survival in ALS patients and why they exhibit enhanced circulating levels of ketone bodies [126,163]. It should be noted that although the utilization of fatty acids may pose an initial bioenergetic benefit, persistent fatty acid oxidation can be detrimental to mitochondrial function due to the increased ROS generated, thus, potentially exacerbating degenerative processes [87]. 

Only a few studies have investigated the status of autophagy in muscle tissue of ALS. Although mutant SOD1 accumulates in the spinal cord of SOD1^G93A^ mice, SOD1 aggregates were not detected in the skeletal muscle of these mice at any disease stage [164]. Thus, it was reported that mutant SOD1-induced autophagic response is much higher in muscle than in the spinal cord of SOD1^G93A^ mice [164].

## 8. Autophagy and Amino Acid Metabolism

Although both autophagy and the ubiquitin–proteasome system can degrade proteins into amino acids, only autophagy is regulated by the concentration of cytosolic amino acids [63]. Therefore, amino acids are not only the end-products of autophagy but also are autophagy modulators [65]. Autophagy modulation mediated by amino acids varies among tissues [165]. Several amino acids, such as glutamine, leucine, arginine, and proline, regulate autophagy by acting as signal molecules [63]. Further, amino acids, such as leucine, tyrosine, glutamine, histidine, tryptophan, proline, methionine, and alanine, have been associated with the suppression of proteolysis in the liver [39]. However, not all these amino acids are equally effective in autophagy regulation [165]. Interestingly, the same amino acids that inhibited hepatic autophagic proteolysis were also known to inhibit proteolysis in other tissues, such as skeletal muscle, heart, and kidney, with leucine being the most effective [166]. Little is known about the amino acid regulation of autophagy in the brain, given that most of the knowledge has originated from studies in the liver and muscle [165].

The crosstalk between autophagy and protein synthesis is mainly mediated by mTORC1 [63]. When amino acids are abundant, mTORC1 inhibits the activity of the complex ULK1-ATG13-FIP200 that is involved in the early steps of autophagosomal biogenesis. By contrast, when amino acids are scarce (or under biochemical blockade using rapamycin treatment), the ability of mTOR phosphorylation is diminished and the complex ULK1-ATG13-FIP200 is quickly dephosphorylated and released to activate the events of autophagosomal formation [165]. Under starvation, the amino acids produced by autophagy provide substrates for gluconeogenesis and ketogenesis in the liver [63]. Further, the molecular mechanisms regulating protein degradation vary during different periods of starvation [63].

### Implications for MND

Studies have demonstrated abnormalities in amino acid metabolism in ALS [167], although most were conducted in the context of glutamate excitotoxicity observed in ALS. Glutamate enzymes, tissue glutamate content, transporter proteins, and postsynaptic receptors were abnormal in ALS models compared to controls [168]. Interestingly, significantly decreased concentrations of valine, isoleucine, leucine, tyrosine, and aspartate were reported in the plasma of ALS patients compared to controls, and a significantly decreased concentration of arginine was observed in patients with a long duration of ALS compared to the patients with a short duration [167]. The clinical state of ALS patients significantly influenced plasma alanine concentration, while other plasma amino acid concentrations were not significantly associated with clinical parameters of the disease [167]. Any direct correlation between the altered amino acid metabolism in ALS and autophagy level currently remains undetermined. 

## 9. Autophagy-Related Metabolic Abnormalities: Sex-Specific Effects

Sex-specific specialization affects body-fat distribution and energy substrate-utilization patterns, where females store more lipids and have higher whole-body insulin sensitivity than males, while males tend to oxidize more lipids than females [169]. These patterns are influenced by the menstrual phase in females, and by nutritional status and exercise intensity in both sexes [169]. Within the context of autophagy, nutrient deprivation decreased mitochondrial respiration, increased autophagosome formation, and produced cell death more profoundly in cultured neurons from males versus females [170]. Starvation-induced neuronal death was attenuated by 3-methyladenine, an inhibitor of autophagy; *ATG7* knockdown using small interfering RNA; or L-carnitine, essential for the transport of fatty acids into mitochondria, all more effective in neurons from males versus females [170]. Overall, during starvation, neurons from males more readily undergo autophagy and die, whereas neurons from females mobilize fatty acids, accumulate triglycerides, form lipid droplets, and survive longer [170]. This study suggests that male neurons are more vulnerable to autophagic death within the context of metabolic perturbation than neurons from females [170], an effect that can be rescued by autophagy inhibition. 

### Implications for MND

ALS is reported to have a male bias with a male:female ratio of 3:1 in incidence [171,172]. ALS prevalence is also higher in men than in women, with a predominance of men with younger disease onset [171,172]. These sex-specific differences have been reported both in studies that included all ALS patients (sporadic and familial) and in familial ALS cases studied separately [173]. Further, a 1.5 male-to-female ratio was reported for most mendelian ALS-related mutant genes, including SOD1 [171]. These sex-specific differences might be attributed to sex steroid androgen hormone regulation where androgen receptor loss was directly associated with motor neuron vulnerability and disease progression in SOD1^G93A^ mice [174]. In addition to this, inherent differential metabolic capabilities that exist within each sex may, at least in part, contribute to these sex-specific effects in ALS. As females can store more lipids and mobilize fatty acids during metabolic stresses, this could offer them an advantage within ALS disease pathophysiology. In this context, it is not surprising that the anti-type 2 diabetic drug metformin treatment gave rise to sex-specific effects on symptom onset and disease progression in female SOD1^G93A^ mice [90]. Metformin caused a reduction in total and LDL cholesterol levels may have interfered with the survival advantage female SOD1^G93A^ mice derive from the mobilization of fatty acids. As male mice already may have activated autophagy in the context of metabolic stress, metformin-induced autophagy has had no effect on their disease course. These results and other sex-specific effects observed with autophagy modulators [120,121] point to the importance of conducting sex-specific analyses when investigating metabolic and autophagy modulators in MND.

## 10. Conclusions

**Figure 1:**
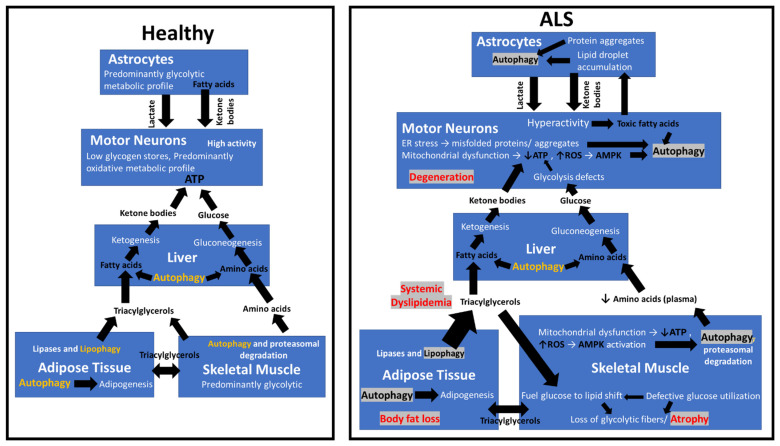
The schematic summarizes the multi-system autophagy-related metabolic dysfunction observed in ALS in comparison to healthy controls. Aberrantly activated autophagy (highlighted in grey) via metabolic deficits in turn may lead to impairments in the rate of autophagy degradation, ↓ decrease, ↑ increase.

Cellular homeostasis is maintained via a complex inter-relationship between energy metabolism and autophagy (for schematics refer to [64]). This association is tightly tuned by various physiological, genetic, and environmental factors. Motor neurons are highly energetic cells that require a vast amount of energy while having limited energy stores where their function and survival require the continuous provision of substantial amounts of nutrients for ATP production (schematics in [84]). Therefore, it is conceivable that during periods of high activity, motor neurons depend heavily on autophagic degradation in addition to conventional glucose metabolism for energy. This may make motor neurons especially vulnerable to perturbations in the autophagy–metabolism inter-relationship, not only at the CNS level but also at the whole-body systemic level (Figure 1). Further studies are required to identify key proteins that modulate the autophagy and metabolism inter-relationship to better understand the trigger points in the disease process and thereby, find therapeutic targets. 

## Figures and Tables

**Table 1:** Few ALS genes categorised according to their encoded protein functions.

Category	Gene	Protein	Function	References
Protein homeostasis	*SOD1*	Superoxide dismutase 1	Antioxidant enzyme-proposed to mediate toxicity in ALS by a gain of function mechanism via protein aggregation	[9,11]
	*SQSTM1*	Sequestosome 1/p62	Protein degradation, autophagy receptor for ubiquitinated cytoplasmic components	[12]
	*OPTN*	Optineurin	Selective autophagy receptor/adaptor that binds and directs polyubiquitinated cargo to autophagosomes, maintains Golgi complex.	[13]
	*VCP*	Valosin Containing Protein	Protein degradation via ubiquitin proteasome system, regulates various steps along the autophagy pathway that promote autophagy induction and autophagosome maturation	[14]
	*UBQLN2*	Ubiquilin 2	Component of the ubiquitin proteasome system	[15]
	*C9orf72*	Chromosome 9 open reading frame 72	Autophagy induction via interaction with the ULK1 complex and Rab GTPases, vesicle trafficking, regulates RNA-splicing	[16,17]
	*VAPB*	VAMP-associated protein B	ER contact protein, modulates biogenesis of autophagosomes by interacting with ATGs	[18]
RNA-binding/metabolism	*TARDBP*	Tar DNA-binding protein 43 kDa (TDP-43)	Binds to DNA and RNA, regulates RNA splicing	[19]
	*FUS*	Fused in sarcoma	RNA metabolism, including transcription, splicing and transport	[20,21]
Cytoskeletal dynamics	*DCTN1*	Dynactin subunit 1	Axonal transport, retrograde transport of vesicles and organelles, attaches cargo to microtubules	[22,23]
	*PFN1*	Profilin 1	Actin regulator, microtubule organization	[24]

**Table 2:** Core autophagy genes and their putative functions.

Yeast Atg Gene	Mammalian Orthologue	Function of Protein	References
*Atg3*	*ATG3*	E2-like enzyme. Part of Atg8/LC3 conjugation system which is required for autophagosome isolation membrane elongation and/or complete closure.	[43]
*Atg4*	*ATG 4A/B/C/D*	LC3/Atg8 C-terminal hydrolase. Deconjugates ATG8 protein	[44]
*Atg5*	*ATG5*	Part of Atg12 conjugation system. The Atg12-Atg5-Atg16(L) dimer is important for Atg8/LC3-phosphatidylethanolamine (PE) conjugation.	[43]
*Atg7*	*ATG7*	E1 enzyme. Part of Atg8/LC3 and Atg12 conjugation systems. Required for Atg8/LC3-PE conjugation (complex present on the outer side of the autophagosome isolation membrane and essential for its proper elongation)	[43,45]
*Atg8*	*ATG8/LC3*	Essential for autophagosome biogenesis/maturation and functions as an adaptor protein for selective autophagy (conjugates to PE on autophagosome membranes).	[46]
*Atg12*	*ATG12*	Forms a complex with ATG5 and ATG16. Assists conjugation of ATG8 to PE	[47]
*Atg13*	*ATG13*	Is part of ULK1 complex. Mediates mTOR signalling	[48,49]
*Atg15*	*ATG15*	Lipase. Involved in autophagic body disintegration (in *Saccharomyces cerevisiae*)	[50]

**Table 3:** Metabolic consequences of autophagy deletion in mice.

Autophagy Gene	Level/Location of Deletion	Consequences	Implication	References
*ATG3*	Constitutive (systemic)	Born developmentally normal but die immediately after birth and show reduced amino acid levels in tissues and plasma during the neonatal starvation period	Autophagy is a critical survival response and required to supply metabolic substrates to bridge gaps in nutrient availability (e.g. neonatal starvation period).	[69]
*ATG5*				[70]
*ATG7*				[71]
*ATG16L1*				[72]
*ATG5*	Neurons	Accumulation of aggregated and ubiquitinated proteins and damaged organelles, motor and behavioural defects, neurodegeneration, and lethality between 1 and 6 months after birth	Essential role of autophagy in post-mitotic tissues and contribution to brain energy metabolism	[68]
*ATG7*				[67]
*ATG5*	Systemic mosaic	Autophagy-deficient hepatocytes show mitochondrial swelling, p62 accumulation, and oxidative stress and genomic damage responses. Lead to benign liver adenomas	Autophagy is important for the suppression of spontaneous tumorigenesis	[73]
*ATG7*	Liver			
*ATG5*	Liver	Mild liver injury characterized by increased apoptosis and compensatory hepatocyte proliferation	Autophagy prevents liver damage	[74]
*ATG7*	Liver	Accumulation of lipid droplets, increased concentration of hepatic triglycerides and cholesterol accompanied with increased liver size.	Autophagy regulates lipid content	[75]
*ATG7*	Adipose tissue	Favours the oxidation of free fatty acids by increasing the proportion of brown adipocytes, which leads to enhanced insulin sensitivity and a lean body mass	Autophagy plays an important role in adipogenesis, and inhibition of autophagy has a unique anti-obesity and insulin sensitization effect	[76]
*ATG7*	Acute whole-body (conditional via tamoxifen induction)	Fatal hypoglycemia upon fasting, susceptibility to infection, extensive liver and muscle damage, neurodegeneration limited survival to 2 to 3 months.	Autophagy dispensable for short-term survival but required to prevent fatal hypoglycemia and cachexia during fasting	[77]
*ATG7*	Hypothalamic agouti-related peptide (AgRP) neurons	Reduces body weight and adiposity without affecting liver mass	Autophagy modulates feeding and energy balance	[78]
*ATG5*	Skeletal muscles	Age-dependent muscle atrophy. Muscle cells exhibit disorganized sarcomeres and accumulation of p62, ubiquitinated proteins, and deformed mitochondria	Confirms homeostatic role of autophagy in skeletal muscle	[79]
*ATG7*				[80]
*ATG7*	Pancreatic β cells	Reduction in β cell mass, hypoinsulinemia, and the accumulation of ubiquitinated proteins, p62, and deformed organelles (i.e., mitochondria and ER)	Basal autophagy is important for maintenance of β cell volume and function	[81,82]
